# Epigenetic Repression of *RUNX2* and *OSX* Promoters Controls the Nonmineralized State of the Periodontal Ligament

**DOI:** 10.3390/genes14010201

**Published:** 2023-01-12

**Authors:** Gokul Gopinathan, Xianghong Luan, Thomas G. H. Diekwisch

**Affiliations:** 1 Center for Craniofacial Research and Diagnosis, Texas A&M University College of Dentistry, Dallas, TX 75246, USA; 2 Department of Oral and Craniofacial Sciences, University of Rochester School of Medicine and Dentistry, 625 Elmwood Avenue, Rochester, NY 14620, USA

**Keywords:** mineralization, histone methylation, *RUNX2*, *OSX*, periodontal

## Abstract

The nonmineralized state of the mammalian periodontal ligament is one of the hallmarks of vertebrate evolution as it provides resilient and nontraumatic tooth anchorage for effective predation. Here we sought to determine how the chromatin state of key mineralization gene promoters contributes to the nonmineralized periodontal ligament in the midst of fully mineralized alveolar bone and cementum anchor tissues. In developing mouse periodontal tissues, *RUNX2* was localized to alveolar bone–lining cells, while *OSX* was localized throughout the periodontal ligament’s soft tissue. Matching RT-PCR amplification data and western blot comparisons demonstrated that the expression of *RUNX2* and *OSX* bone mineralization transcription factors was at least 2.5-fold elevated in alveolar bone osteoblasts versus periodontal ligament fibroblasts. ChIP enrichment data along the *RUNX2* and *OSX* promoters revealed increased H3K4me3 marks in alveolar bone osteoblasts, while H3K9me3 and H3K27me3 marks were elevated in periodontal ligament fibroblasts. In support of an epigenetic mechanism responsible for the inhibition of mineralization gene expression in periodontal progenitors, histone methylation inhibitors DZNep and Chaetocin reactivated *RUNX2* and *OSX* expression in periodontal progenitors and increased alkaline phosphatase and Alizarin Red, while the in vivo application of DZNep in rat maxillae resulted in aberrant mineralization in the periodontal ligament and a narrowing of the nonmineralized periodontal space. Together, these studies demonstrate that the nonmineralized state of the mammalian periodontal ligament is controlled by an epigenetic regulation of the *RUNX2* and *OSX* key mineralization gene promoters.

## 1. Introduction

The commitment of progenitor cells to form mineralized extracellular matrices is a complex, multiple-level decision-making process that involves the transcriptional and epigenetic control of cell fate and gene expression, the determination of extracellular matrix protein composition, posttranslational modifications of secreted proteins, and the regulation of the ion balance through mineral homeostasis. Key aspects of multiscale mineralized tissue control include (i) the transcriptional regulation of mineralized tissue lineage commitment through *OSX* (Osterix) and *RUNX2* [1,2,3,4]; (ii) the secretion of mineralized tissue extracellular matrices rich in collagen, bone sialoprotein (BSP), and proteoglycans [2,3]; (iii) enzymatic processing important in the initiation of mineralization through tissue-specific alkaline phosphate (TNAP) [5,6] and phosphate orphan 1 (PHOSPHO1) [7,8]; and (iv) the regulation of mineral homeostasis via the hydrolysis of the mineralization inhibitor pyrophosphate and the control of calcium/phosphate ratios [9]. Although much is known about the downstream regulation of tissue mineralization, our understanding of the epigenetic regulation of mineralized tissue lineage commitment and transcriptional control of mineralized extracellular matrix secretion is still in its infancy.

One of the most intriguing examples of precise mineralization control is the mammalian periodontal ligament, a tissue in which a nonmineralized periodontal ligament is bordered by two mineralized tissues: root cementum and alveolar bone. The width of the nonmineralized periodontal ligament in proximity to the mineralized tooth root surface is highly consistent over the entire length of the tooth root and changes only slightly when teeth move thanks to drift or orthodontic forces [10,11,12]. Although it has been speculated that *Wnt* signals may regulate mineral homeostasis in the periodontal region [13,14], little is known about the chromatin state on key mineralization gene promoters *RUNX2* and *OSX* insofar as how they affect mineralized tissue lineage commitment and matrix secretion.

The epigenetic state of gene promoters as it relates to the regulation of gene expression is tightly controlled by a number of histone modifications, including the methylation of lysine and arginine and the acetylation of lysine amino acids [15,16]. Whereas histone acetylation commonly results in the activation of gene expression, histone methyl marks are associated either with upregulation or gene repression, depending on the site and degree of methylation [16]. Apart from regulating chromatin structure, these modifications serve as dynamic binding platforms for various effector molecules that fine-tune gene transcription. For example, the active H3K4me3 mark is recognized by plant homeodomain (PHD) finger proteins, which in turn recruit activating chromatin modifiers such as histone acetyl transferases (HATs) and histone deacetylases (HDACs), while H3K9me3 marks are specifically recognized by heterochromatin protein 1 (HP1), leading to chromatin compaction and gene repression [17]. A number of studies have documented that epigenetic modifications influence the gene expression of key mineralization genes during differentiation [18,19]. For example, the transcriptional activity of *RUNX2* is associated with distinct chromatin remodeling events on the proximal region of the P1 promoter during osteoblast differentiation characterized by the presence of DNaseI-hypersensitive sites and an increase in histone H3 and H4 acetylation during the early stages of mesenchymal differentiation [20]. *RUNX2* also regulates target gene expression through its direct interaction with SUV39H1 proteins and its ability to form a corepressor complex with HDAC1 [21]. The transcriptional activation of the other key mineralization transcription factor, *OSX*, was associated with increased recruitment in the histone acetylase, p300, and decreased H3K9 methylation at its promoter [22]. In addition, the promoter of the downstream gene, IBSP, is regulated by chromatin modifications mediated through interactions between *OSX* and the histone demethylase NO66 [23]. Moreover, the histone demethylase KDM6B promotes osteogenic commitment by removing H3K27me3 marks on the promoters of the *OSX* transcription factor and the extracellular matrix genes, *OCN* and *SPP1* [24]. It has also been demonstrated that the removal of H3K27me3 marks on the *RUNX2* and *OSX* promoters by the histone demethylase JMJD3 caused the transcriptional activation of *RUNX2* and *OSX* and the resulting osteoblast differentiation [25].

In the present study, we focus on the periodontal complex as a model system to understand epigenetic dynamics involved in mineralized state control of periodontal progenitors. We hypothesize that having epigenetic control of the transcriptional state of key mineralization gene promoters is a key requirement in the cascade of events responsible for tight the spatial definition of the nonmineralized periodontal ligament in mammals. Our study focuses on the effect of key histone modifications, H3K9me3 and H3K27me3, as repressive marks and H3K4me3 as an active mark on two major mineralized tissue lineage commitment genes, *RUNX2* and *OSX*. Our data reveal a direct relationship between the epigenetic states of key mineralization transcription factor promoters and mineralized extracellular matrix secretion as a promoter-level explanation for the repression of mineralization in the periodontal ligament.

## 2. Materials and Methods

### 2.1. Isolation of Human Dental Progenitors

Human teeth extracted for orthodontic reasons from healthy patients (ranging from 12 to 15 years of age) were used for the isolation of dental progenitor populations. All extraction procedures were performed in accordance with the human subject protocol approved by Texas A&M University Institutional Review Board. PDL and AB progenitors were isolated by using collagenase/dispase treatment after carefully dissecting the corresponding tissues from developing tooth organs, as described previously [26]. Progenitors were maintained in complete media (1X DMEM containing 10% FBS, 2 mM L-Glutamine, 100 U/mL Penicillin and 100 µg/mL of streptomycin) at 37 °C in 5% CO_2_. All experiments were performed with early passage cells (P3-6).

### 2.2. Tissue Collection and Histology

Mandibles were dissected from 10-day-old wild-type C57/BL6 mice and fixed in 10% neutral buffered formalin at 4 °C. Decalcification was carried out in 4.5% EDTA, 0.45% NaOH, and 1% formalin at 4 °C and processed for paraffin embedding. Further, 5 µm sections were mounted onto glass slides and dried overnight. Immunohistochemical analysis was performed on the samples as follows. Sections were first deparaffinized, rehydrated, and treated with 3% hydrogen peroxide in methanol. Antigen retrieval was achieved by incubation with 1X Trypsin-EDTA pH 8.0 at 37 °C for 30 min. After blocking for nonspecific activity with 5% BSA, the samples were incubated overnight at 4 °C with an anti-*OSX* (ab22552) and anti-*RUNX2* (ab76956) antibody (1:50; Abcam, Cambridge, MA, USA) in 1X PBS containing 0.5% Tween 20. Subsequent steps were carried out as per instructions in the HistoMouse-Plus Kit (Life Technologies, Grand Island, NY, USA). Briefly, the sections were washed with 1XPBST and incubated with a biotinylated secondary antibody, followed by a streptavidin-peroxidase conjugate. Protein expression was visualized using the AEC (red) Substrate Kit (Life Technologies) and counterstained with Hematoxylin (Life Technologies). Trichrome staining was carried out on paraffin sections using Masson’s trichrome stain kit (MilliporeSigma, St. Louis, MO, USA).

For nondecalcified ground sections, formalin-fixed maxillae were dehydrated in a graded ethanol series, followed by infiltration with Technovit 7200VLC resin (EXAKT Technologies, Oklahoma City, OK, USA). Subsequently, they were embedded in resin, and thin sagittal sections (20 µm) were obtained following the EXAKT cutting and grinding technique (Donath). Von Kossa procedure for calcium detection was carried out by treating plastic-embedded tissue sections with 5% silver nitrate solution, as described previously [27]. Stained sections were visualized and imaged on a Leica DMRX microscope (Leica, Nuhsbaum, Inc., McHenry, IL, USA).

### 2.3. Chromatin Immunoprecipitation Analysis

ChIP analysis was performed as previously described [28], with some modifications on control and 7-day or 14-day induced PDL and AB progenitors. Briefly, PDL and AB progenitors grown on 150 mm dishes were crosslinked with 1.1% formaldehyde for 10 min and the reaction quenched with 125 mM glycine. The cells were then harvested, pooled, flash frozen, and stored at −80 °C. For each immunoprecipitation, 1 × 10^6^ cells were first incubated in lysis buffer 1 (50 mM HEPES-KOH, pH 7.5, 140 mM NaCl, 1 mM EDTA, 10% glycerol, 0.5% NP-40, 0.25% Triton X-100, 1× protease inhibitors) to isolate nuclei that were resuspended in lysis buffer 2 (10 mM Tris-HCl, pH 8.0, 100 mM NaCl, 1 mM EDTA, 0.5 mM EGTA, 0.1% Na-deoxycholate, 0.5% *N*-lauroylsarcosine, 1× protease inhibitors). The chromatin was then sheared to a size of 300 bp–1 kb using a cup horn sonicator (Q Sonica, Newtown, CT, USA) and clarified at 12,000 rpm for 10 min. An input fraction corresponding to 5% of the starting material was kept aside for ChIP normalization. Immunoprecipitation reactions were performed by incubating equal amounts of sheared chromatin with 100 µL of DynaI beads (Life Technologies) prebound to 10 µg of antibody against each of the histone modifications: H3K4me3 (ab8580), H3K9me3 (ab8898), and H3K27me3 (ab6002) (Abcam). After overnight incubation at 4 °C, beads were washed 5 times with 1X RIPA buffer (50 mM HEPES-KOH, pH 7.5, 500 mM LiCl, 1 mM EDTA, 1.0% NP-40, 0.7% Na-deoxycholate) and once with 1X TE (10 mM Tris-HCl pH 8.0, 1 mM EDTA pH 8.0). Elution of protein-DNA complexes was carried out by incubating beads in elution buffer (50 mM Tris-HCl, pH 8.0, 10 mM EDTA, 1.0% SDS) followed by crosslink reversal at 65 °C overnight. DNA purification was carried out using DNA Clean & Concentrator kit (Zymo Research, Irvine, CA, USA), following manufacturer’s guidelines. Real-time quantitative polymerase chain reaction (PCR) was performed with SYBR green Master Mix (Life Technologies) on a Bio-Rad C1000 Thermal Cycler (Bio-Rad, Hercules, CA, USA), using specific primer pairs against promoters of *RUNX2* and *OSX* (Table S1). Amplification data for each primer pair are normalized to input chromatin, and histone modification enrichment data are presented after subtracting the values of corresponding negative controls (beads alone). Graphed data are from four or more independent ChIP reactions performed for each condition in each cell type.

### 2.4. Osteogenic Induction and Inhibitor Treatment of Dental Progenitors

PDL and AB progenitors were seeded at a density of 26,000 cells/cm^2^ and cultured overnight before treatment. Osteogenic differentiation was carried out by culturing cells with osteogenic induction medium (complete media supplemented with 50 µg/mL ascorbic acid 2-phosphate, 10 mM β-glycerophosphate, and 10 mM dexamethasone; all from MilliporeSigma). Cells were harvested after 7 or 14 days for ChIP assays, after 7 days for alkaline phosphatase assays, and after 21 days for Alizarin Red S staining assays. The induction media were changed every alternate day. Progenitor cells grown in regular culture media were used as a reference group (day 0). For epigenetic inhibitor treatment, PDL progenitors were cultured in the presence of H3K27me3 inhibitor, DZNep (MilliporeSigma) (5 µM, 10 µM, or 20 µM), and H3K9me3 inhibitor, Chaetocin (MilliporeSigma) (5 nM, 10 nM, or 20 nM), for 48 h, and the total RNA was isolated as described below.

### 2.5. RNA Isolation and Semiquantitative Real-Time PCR

Total RNA was isolated using the RNeasy PLUS Mini Kit (Qiagen, Valencia, CA, USA), according to manufacturer’s instructions. Here 1 µg of total RNA was used for cDNA synthesis using SuperScript III Reverse Transcriptase (Life Technologies). The mRNA expression levels were quantified with sequence-specific primers (Table S1), using SYBR green Master Mix (Life Technologies) and the Bio-Rad C1000 Thermal Cycler (Bio-Rad). Reaction conditions are as follows: initial activation for 20 s at 95 °C, 40 cycles of 3 s at 95 °C and 30 s at 60 °C, followed by a melt curve analysis. GAPDH was used as an internal control for normalization. Reproducibility of the results was confirmed by performing the analysis in triplicates for at least four independent experiments. Relative transcript levels were calculated using the 2^−∆∆Ct^ method [29], and values were represented as mean expression ± standard deviation (SD).

### 2.6. Western Blot Analysis

Whole cell lysates were prepared from early passage PDL and AB progenitors by using 1X RIPA buffer containing protease inhibitors (Roche, Indianapolis, IN, USA). Here 50 µg of total protein was subjected to SDS-polyacrylamide gel electrophoresis and transferred to a PVDF membrane (EMD Millipore, Billerica, MD, USA). After blocking for nonspecific binding, the membranes were incubated with antibodies against *RUNX2* (ab76956), *OSX* (ab22552), IBSP (ab33022) and β-ACTIN (ab3280) (antibodies were from Abcam and used at a dilution of 1:1000). Immune complexes were detected using goat antimouse or goat antirabbit HRP conjugated secondary antibody (Santa Cruz Biotechnology, Dallas, TX, USA) and enhanced Chemiluminescence reagents (Pierce Biotechnology, Rockford, IL, USA).

### 2.7. Alkaline Phosphatase Assay and Alizarin Red Staining

Alkaline phosphatase activity was determined by staining methanol-fixed control and -treated cells with NBT/BCIP solution (Roche) for 12 min. Mineral deposits were detected by fixing cells with cold methanol and staining with 1% Alizarin Red S solution (MilliporeSigma). Phase-contrast images were obtained on an inverted microscope (Leica). Image analysis and densitometry analysis were carried out using ImageJ software (NIH).

### 2.8. Inhibitor Treatment in Rat Maxilla Model

Adult Sprague Dawley rats (weight, 125–175 g) obtained from Charles River Laboratories (Wilmington, MA, USA) were housed in a temperature- (20–23 °C) and humidity-controlled environment (50%) on a regular light–dark schedule. All experimental procedures were approved by the Institutional Animal Care and Use Committee of Texas A&M University, Health Science Center. A periodontal pocket was created by gently widening the periodontal space on the palatal side of maxillary molar tooth using a sterile curette, ensuring that only minimal damage was caused to the surrounding bone and gingiva. The surgical site encompassed the palatal side of the distolingual and mesiolingual roots of the first molar. Sterile collagen sponge (approx. 1 mm^3^) soaked in 20 µM DZNep inhibitor (MilliporeSigma, dissolved in sterile saline) was implanted into the periodontal pocket by using sterile tweezers. Control molars were similarly treated with a saline-soaked collagen sponge. Postsurgery, rats were maintained on a regular diet and monitored daily for infections. After 3 weeks, maxillae were dissected out and fixed in 10% formalin for Micro-CT analysis. The treatment was repeated four times with 3–4 animals in each experimental group.

### 2.9. Micro-CT Analyses

Rat maxillae in 0.5% formalin were scanned on a desktop µCT (µCT-20, Scanco Medical AG, Bruttisellen, Switzerland) at a resolution of 10 µm, a voltage of 55 kVp, and an integration time of 800 ms. Three-dimensional reconstructions were generated from two-dimensional images using Scanco analysis software. To visualize PDL space around the first molar roots, the region of interest (ROI) was manually defined on individual CT slices. The ROI included the entire root, the surrounding ligament space, and a small area of adjacent alveolar bone. Next, the contoured slices were thresholded (lower threshold—0; upper threshold—300) to selectively reveal the PDL space and subjected to 3D reconstruction and morphometric analysis. PDL volume measurements were performed on the distobuccal root away from the lingual side to minimize any surgery-induced artifacts.

### 2.10. Statistical Analysis

Data were analyzed using the GraphPad Prism 8.0 software and are graphed as mean ± SD (standard deviation). Difference between two groups was analyzed using the Student’s *t*-test and considered statistically significant at *p* < 0.05. Levels of statistical significance are denoted as follows: * *p* < 0.05, ** *p* < 0.01, *** *p* < 0.001.

## 3. Results

### 3.1. Key Mineralization-Related Transcription Factors, RUNX2 and OSX, Were Highly Expressed in AB Progenitors Compared with PDL Progenitors

**Figure 1:**
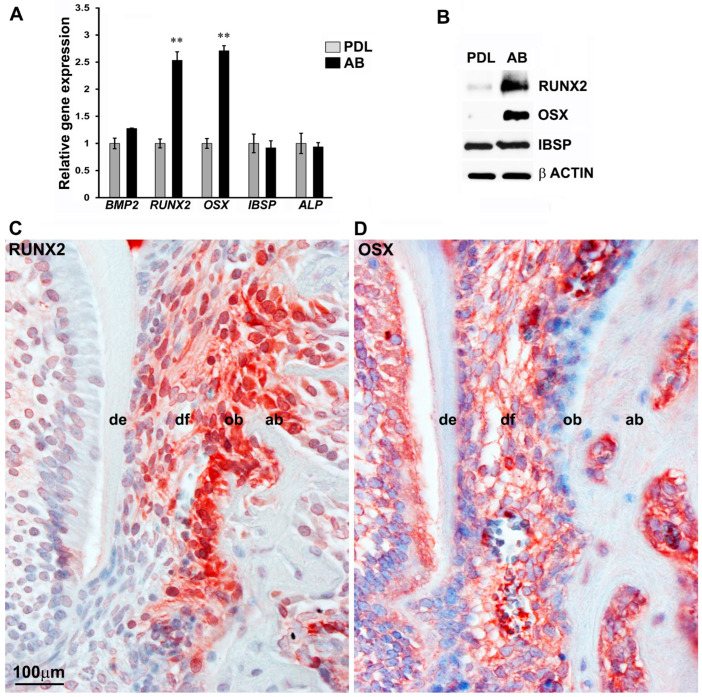
Master osteoblast transcription factors *RUNX2* and *OSX* are highly expressed in AB progenitors compared with PDL cells. (**A**) Comparison of transcript levels for mineralization-related genes *BMP2*, *RUNX2*, *OSX*, *IBSP*, and *ALP* between PDL and AB progenitors. GAPDH levels were used to normalize gene expression. *RUNX2* and *OSX* levels were expressed greater than 2-fold in AB cells compared with PDL cells (n = 4–6). Data are graphed as mean ± SD. (** *p* < 0.01). (**B**) Western blot analysis for *RUNX2*, *OSX*, and *IBSP* expression in PDL and AB progenitors. β ACTIN was used as a loading control. Immunohistochemical analysis for *RUNX2* (**C**) and *OSX* (**D**) expression in a 10-day-old developing mouse molar. Note the high expression observed for both proteins in osteoblasts within alveolar bone. de = dentin; df = dental follicle; ob = odontoblasts; ab = alveolar bone osteoblasts.

To determine whether a complex transcriptional state distinguishes the lineage commitment of craniofacial progenitors, we focused on the *RUNX2* and *OSX* transcription factors as essential regulators of osteoblast lineage commitment. We compared the transcript levels of both *RUNX2* and *OSX* between the less-differentiated periodontal ligament fibroblasts (PDL) and the osteoblast lineage-committed alveolar bone (AB) cells. PDL and AB progenitor cells represent contrasting stages of lineage commitment among the craniofacial progenitors, and both cell types can give rise to highly differentiated osteoblasts, making them well suited to study the transcriptional control of early mineralization regulators. RT-PCR analysis on early passage progenitors indicated that the transcript levels of both *RUNX2* and *OSX* were significantly upregulated (>2-fold) in AB cells compared with PDL cells (Figure 1A). Our analysis did not detect any significant difference in the expression level of the bone morphogenetic family member, *BMP2*, and the early mineralization marker, *ALP*, between PDL and AB cells (Figure 1A). Transcript levels of the bone-related marker gene, IBSP, were also found to be similar between PDL and AB progenitors in our RT-PCR assays (Figure 1A). Further confirmatory analysis of key mineralization marker gene expression was carried out by western blot analysis on lysates from early passage human PDL and AB progenitors. Our analysis indicated significantly elevated levels of *RUNX2* and *OSX* proteins in AB progenitors, whereas they were barely detectable in PDL lysates (Figure 1B). As expected, there was no difference observed for IBSP protein levels among the progenitor types (Figure 1B).

To further access the localization of early mineralization regulators within the context of craniofacial tissues, *RUNX2* and *OSX* proteins were evaluated in the developing mouse molar model. An immunohistological analysis on 10-day-old mouse molars revealed a highly specific expression pattern for *RUNX2* (Figure 1C) and *OSX* (Figure 1D) in alveolar bone osteoblasts. In addition, *RUNX2* was also expressed in the odontoblasts (Figure 1C), while *OSX* expression was detected in the developing dental follicle (Figure 1D). In essence, the expression levels of the key bone transcription factors, *RUNX2* and *OSX*, were higher in AB cells compared with PDL cells.

### 3.2. PDL Cells Were Distinguished from AB Progenitors by a Repressive Chromatin State on RUNX2 and OSX Promoters

**Figure 2:**
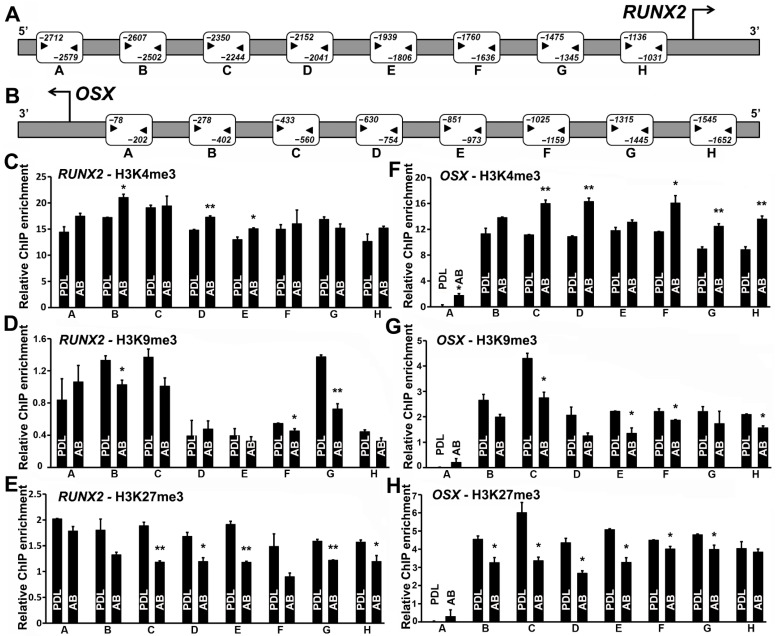
*RUNX2* and *OSX* promoters are characterized by repressive histone modifications in PDL progenitors. Human *RUNX2* promoter (**A**) and *OSX* promoter (**B**). Region upstream to the transcription start site (TSS) was probed for histone H3K4me3, H3K9me3, and H3K27me3 enrichment with 8 primer pairs, as indicated. Primer binding sites are indicated alongside the regions amplified for both *RUNX2* and *OSX* promoters. ChIP-qPCR-based histone H3K4me3 (**C**,**F**), H3K9me3 (**D**,**G**), and H3K27me3 (**E**,**H**) enrichment at the regions indicated on *RUNX2* and *OSX* promoters, respectively. Note the higher levels of repressive histone modifications (H3K9me3 and H3K27me3) on *RUNX2* and *OSX* promoters in PDL when compared with AB cells. The active histone modification H3K4me3 was preferentially enriched on *RUNX2* and *OSX* promoters in AB cells when compared with PDL cells (**C**,**F**). ChIP analyses were independently performed with each primer pair, and representative enrichment plots are shown (n = 4–5). Enrichment values are graphed as mean ± SD (* *p* < 0.05, ** *p* < 0.01).

Our expression analysis at the mRNA and protein levels documented significantly higher levels of *RUNX2* and *OSX* transcription factors in AB progenitors compared with PDL cells (Figure 1A,B). In order to mechanistically explain the differential transcriptional control of *RUNX2* and *OSX* expression between PDL and AB cells, we decided to focus on epigenetic markers as a possible mechanism that can influence gene expression. Specifically, we hypothesized that the native chromatin states existing at *RUNX2* and *OSX* promoters in these cell types could be predictive of their mineralized/nonmineralized states. In order to gain further insights in this regard, we performed a comprehensive analysis of histone modification occupancy on two repressive histone marks (H3K9me3 and H3K27me3) and an active histone mark (H3K4me3) at the promoters of *RUNX2* and *OSX* in PDL and AB progenitors. These histone methylation markers were chosen on the basis of our previous study establishing H3K4, H3K9, and H3K27 methylation as key epigenetic determinants of lineage and differentiation potential in dental follicle and dental pulp cells [28]. A region of approximately 2.7 kb and 1.6 kb upstream to the transcription start site of *RUNX2* and *OSX* promoters, respectively, were probed with primer pairs targeting eight regions for the enrichment of H3K4me3, H3K9me3, and H3K27me3 histone modifications (Figure 2A,B).

Our analysis in general indicated a higher enrichment for the active H3K4me3 histone marks on both *RUNX2* and *OSX* promoters in the case of AB progenitors compared with PDL (Figure 2C,F). Specifically, in the case of *RUNX2*, regions B, D, and E were significantly enriched for H3K4me3 marks in AB cells (Figure 2C), while in the case of *OSX*, all promoter regions tested, excluding regions B and E (Figure 2F), displayed significantly higher enrichment for this mark in AB progenitors when compared with PDL cells. ChIP analysis for the repressive histone modifications (H3K9me3 and H3K27me3) displayed a general trend of higher enrichment levels for both histone marks on *RUNX2* and *OSX* promoters in PDL progenitors, indicating a higher state of repression for both proteins when compared with AB progenitors (Figure 2D–H). *RUNX2* promoter regions B, F, and G exhibited significant enrichment for H3K9me3 modifications in PDL progenitors when compared with AB progenitors, while in the case of the *OSX* promoter, we observed a higher and significant enrichment for this histone modification mark at regions C, E, F, and H in PDL cells compared with AB progenitors (Figure 2D,G). Promoter ChIP analysis for the H3K27me3 modification displayed consistently increased enrichment across the entire promoter of *RUNX2* and *OSX* in PDL progenitors compared with AB cells. Promoter regions C, D, E, G, and H of *RUNX2* predominantly featured as regions harboring significantly higher levels of H3K27me3 in PDL cells compared with AB cells (Figure 2E). In the case of *OSX*, all promoter regions probed in AB progenitors demonstrated significantly lower levels of H3K27me3, with the exception of regions A and H, when compared with PDL cells (Figure 2H). Hence, a repressive chromatin state dictated by histone modifications might be responsible for the overall lower expression of *RUNX2* and *OSX* in PDL cells.

### 3.3. Osteogenic Induction Results in a Gradual Increase in H3K4me3 Marks on the Alveolar Bone Osteoblast RUNX2 Promoter versus a Steep Increase and Decline on the PDL Progenitor RUNX2 Promoter

**Figure 3:**
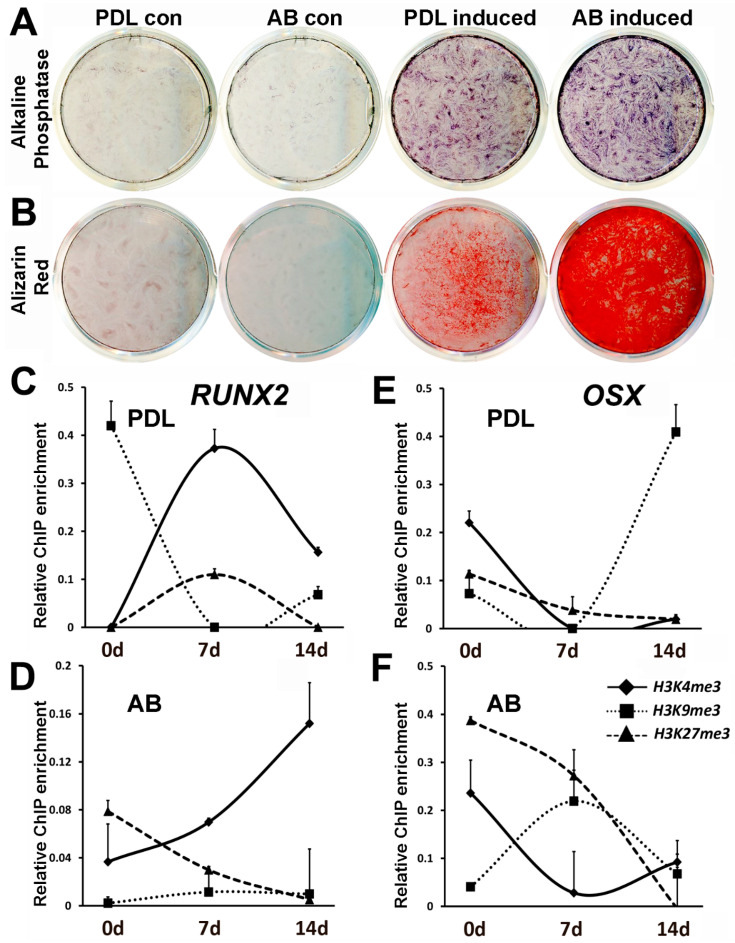
Comparison of PDL and AB progenitor mineralization potential and histone modification dynamics at *RUNX2* and *OSX* promoters upon osteoinduction. (**A**) Alkaline phosphatase activity in PDL and AB progenitors subjected to mineralizing conditions for 7 days. (**B**) Calcium deposition analyzed by Alizarin Red staining in PDL and AB cells after 21 days of induction. (**C**–**F**) Histone dynamics for *RUNX2* and *OSX* promoters in PDL and AB progenitors under mineralizing conditions after 7 and 14 days. Changes in histone H3K4me3, H3K9me3 and H3K27me3 enrichment levels are depicted as scatter plots. ChIP analysis for *RUNX2* promoter in (**C**) PDL cells and in (**D**) AB cells. ChIP analysis for *OSX* promoter in (**E**) PDL cells and in (**F**) AB cells. ChIP data are representative of independent experiments with similar results (n = 4–5). Enrichment values are graphed as mean ± SD.

Although the periodontal ligament remains in a nonmineralized state throughout the life of mammals, the periodontal region provides an essential reservoir for osteoblast and cementoblast stem cells for the continued remodeling of the periodontium [30,31]. In order to compare the level of mineralized lineage differentiation between PDL and AB progenitors, cells were subjected to osteoinductive conditions in vitro. Osteoinductive conditions have been successfully used to induce differentiation of dental progenitors in vitro, resulting in higher osteoblast marker expression and increased calcium deposits [32,33,34,35]. Our analysis indicated that after 7 days of induction under osteogenic conditions, AB progenitors significantly upregulated alkaline phosphatase activity compared with PDL cells (Figure 3A). Similarly, Alizarin Red assay demonstrated a massive amount of calcium deposition in AB progenitors compared with PDL cells after 21 days of osteoinduction (Figure 3B). These results demonstrate that although PDL cells have an ability to produce mineralized nodules upon osteoinduction, the response was rather limited compared with AB progenitors.

We next asked whether the mineralization response observed in PDL and AB cells are accompanied by changes in histone modifications of key bone marker genes. We again focused on mineralization transcription factors *RUNX2* and *OSX* for this assay on the basis of our previous findings’ demonstrating a higher level of epigenetic repression on promoters of *RUNX2* and *OSX* in PDL cells compared with AB cells. A ChIP analysis was carried out on PDL and AB progenitors after 7 and 14 days of osteogenic differentiation using antibodies against H3K4me3, H3K9me3, and H3K27me3 histone modifications. Our analysis indicated that *RUNX2* promoter regulation was mediated mostly through H3K4me3 marks, while the *OSX* promoter was regulated by complex and dynamic changes in histone modifications (Figure 3C–F). Mineralization induction led to a sustained increase in the levels of the active H3K4me3 mark on the *RUNX2* promoter after 7 and 14 days in AB progenitors (Figure 3D), while in the case of PDL cells, there was an initial increase followed by decreased enrichment for H3K4me3 mark after 7 days of induction (Figure 3C). Osteogenic induction was accompanied by a steep decline in the repressive H3K9me3 marks on the *RUNX2* promoter in PDL cells (Figure 3C).

The histone modification changes on the *OSX* promoter upon osteoinduction were far more intricate, with a dominant role for repressive histone modifications H3K9me3 and H3K27me3. The predominant trend in AB cells was a gradual and substantial decrease in enrichment for H3K27me3 after 7 and 14 days of osteoinduction (Figure 3F), while in the case of PDL cells, the decrease in enrichment for repressive histone marks was not prominent (Figure 3E). Interestingly, the level of enrichment for the active H3K4me3 mark at the *OSX* promoter decreased in both PDL and AB cells after 7 days of mineralization induction (Figure 3E,F).

### 3.4. Reactivation of RUNX2 and OSX Gene Expression by Histone Methylation Inhibitors Caused an Enhanced Mineralization Response in PDL Cells

**Figure 4:**
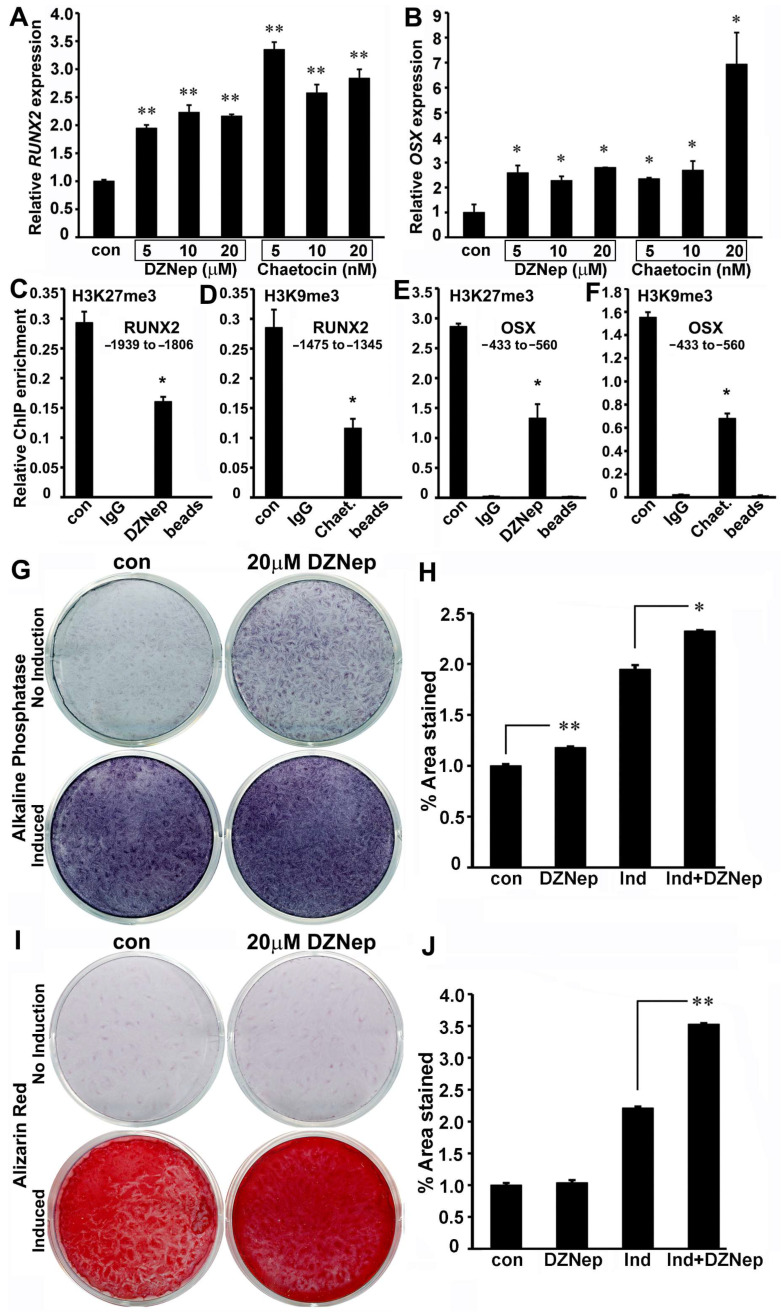
Epigenetic reactivation of *RUNX2* and *OSX* gene expression led to increased mineralization in PDL progenitors. (**A**,**B**) PDL cells were treated with inhibitors against H3K27me3 (DZNep) and H3K9me3 (Chaetocin) for 48 h, and transcript levels of *RUNX2* (**A**) and *OSX* (**B**) were analyzed by RT-PCR. Transcript levels for both genes were upregulated upon inhibitor treatment in PDL cells, with Chaetocin treatment leading to higher expression levels. (**C–F**) ChIP analysis in DZNep and Chaetocin-treated PDL cells for H3K27me3 and H3K9me3 enrichment on (**C**,**D**) *RUNX2* and (**E**,**F**) *OSX* promoters, respectively. Untreated cells were used as controls, and beads-alone immunoprecipitations were used to determine ChIP specificity and background. Promoter regions analyzed are indicated for respective enrichment plots. RT-qPCR data and ChIP experiments are representative of independent experiments with similar results (n = 4–6 for RT-PCR assays; n = 4–5 for ChIP assays). (**G**) Alkaline phosphatase activity in PDL progenitors treated with 20 µM DZNep for 7days with or without osteogenic induction. Untreated cells served as controls. (**H**) Representative quantitation of *ALP* activity after DZNep treatment in PDL cells. (**I**) Alizarin Red assay for calcium deposition in PDL cells treated with DZNep as in (**G**) after 21 days of culture. (**J**) Representative quantitation of Alizarin Red staining intensity after DZNep treatment in PDL cells. All data are presented as mean ± SD. (* *p* < 0.05, ** *p* < 0.01). µM, micromolar; nM, nanomolar.

Next, we analyzed whether high levels of repressive histone modifications H3K9me3 and H3K27me3 were indeed responsible for the decreased expression of the *RUNX2* and *OSX* transcription factors in PDL cells. PDL progenitors were treated with histone methylation inhibitors DZNep and Chaetocin, which decreased the global levels of H3K27me3 and H3K9me3 histone modifications, respectively [36,37]. An RT-PCR analysis indicated that treating PDL cells with DZNep for 48 h led to a greater than 2-fold increase in *RUNX2* and *OSX* mRNA levels (Figure 4A,B). Chaetocin treatment, on the other hand, resulted in much higher upregulation (>3-fold) for transcript levels of *RUNX2* and *OSX* in PDL cells, with 20 nM Chaetocin treatment bringing about a 7-fold increase in *OSX* levels in PDL cells (Figure 4A,B).

We next wanted to determine whether histone methylation inhibitors directly modulated the chromatin state of *RUNX2* and *OSX* promoters to increase their expression in PDL cells. A ChIP analysis on PDL cells treated with DZNep and Chaetocin for 72 h demonstrated a dramatic decrease in levels of H3K27me3 and H3K9me3 histone modification, respectively, on both *RUNX2* and *OSX* promoters compared with untreated cells (Figure 4C–F). Although H3K9me3 inhibition was able to elicit a higher expression of *RUNX2* and *OSX* transcripts, long-term treatment (>72 h) with Chaetocin led to cellular toxicity in PDL cells—hence the DZNep inhibitor was used for subsequent studies. In order to determine the effect of histone methylation inhibitor–mediated *RUNX2* and *OSX* activation on extracellular matrix secretion, PDL cells were treated with 20 µM DZNep, with or without mineralization induction. Our analysis indicated significantly elevated alkaline phosphatase activity (*p* < 0.05) in DZNep-treated PDL cells after 7 days of osteogenic induction compared with nontreated induced cells (Figure 4G,H). Interestingly, DZNep treatment alone was able to significantly elevate *ALP* activity (*p* < 0.01) in PDL cells in the absence of mineralization induction (Figure 4F,H). Next, the ability of PDL cells to give rise to mineralized nodules under osteogenic induction was assayed using Alizarin Red staining after 21 days of osteogenic induction. While treatment with mineralizing media was able to drastically elevate calcium deposition in PDL cells, the addition of the DZNep inhibitor further elevated this response (Figure 4I). Quantification of Alizarin Red staining levels confirmed that the increased calcium deposition in DZNep-treated PDL cells were statistically significant compared with controls (*p* < 0.01) (Figure 4J). Although DZNep alone was able to elevate *ALP* activity, it was not sufficient to initiate extracellular calcium deposition in PDL cells in the absence of osteogenic induction (Figure 4I,J).

### 3.5. H3K27me3 Inhibition in a Rat Maxilla Model Resulted in Aberrant Periodontal Ligament Mineralization and Decreased Width of the Nonmineralized Periodontal Region

**Figure 5:**
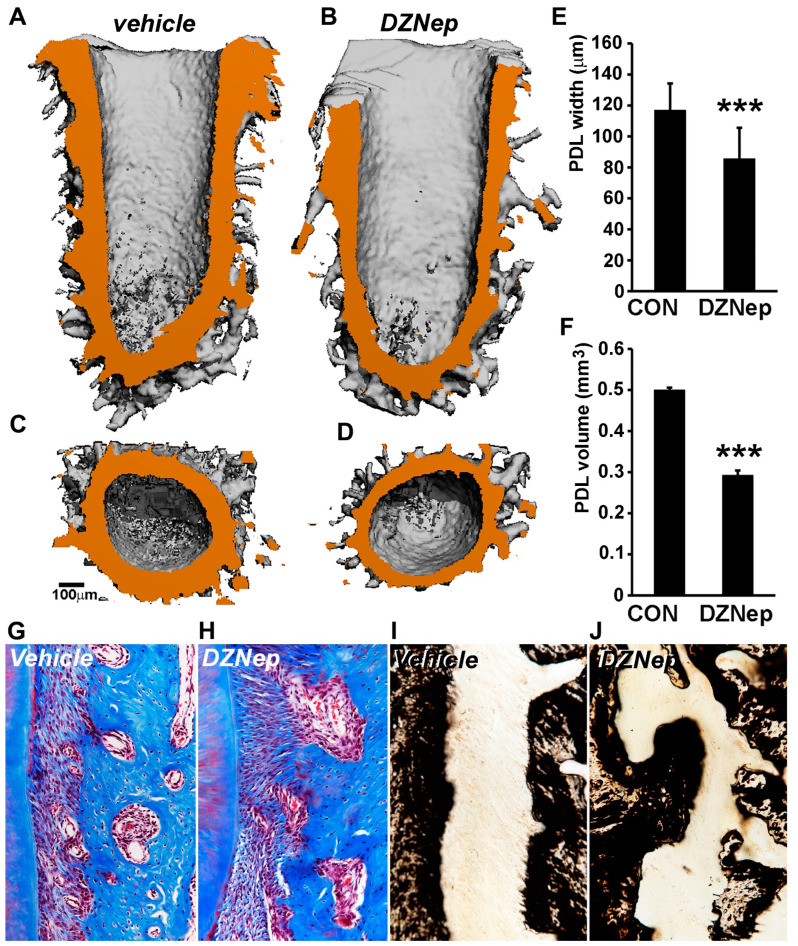
Aberrant mineralization and decrease in periodontal ligament width in an in vivo rat maxilla model treated with H3K27me3 inhibitor DZNep. (**A**–**F**) The µCT analyses of control and inhibitor-treated rat maxilla. (**A**) Representative sagittal section derived from 3D reconstruction of distobuccal root in vehicle-treated samples. (**B**) Representative sagittal section from DZNep inhibitor-treated root samples. PDL space is rendered orange for easy visualization. Note the decreased PDL width in inhibitor-treated samples compared with vehicle-treated samples. Transverse view through distobuccal root at the mid-root region in (**C**) vehicle-treated and (**D**) DZNep-treated samples. (**E**) Quantification of average PDL width of distobuccal root in control/vehicle (con) and DZNep-treated samples. Measurements were performed on Image J software (NIH) along the entire length of the PDL space. More than 50 measurements were performed for each sample (n = 10). (**F**) Comparison of total PDL volume (tissue volume) of distobuccal root in control/vehicle (con) and DZNep inhibitor-treated samples. Additionally, 3–4 volume measurements were performed for each sample (n = 10). (**G**,**H**) Representative histomicrograph of Masson’s trichrome staining for vehicle (control) and DZNep-treated rat maxillary first molar. (**I**,**J**) Von Kossa staining for mineral deposits in vehicle (control) and DZNep-treated samples. Note the mineralized outgrowths in inhibitor-treated samples. Measurements are graphed as mean ± SD. (*** *p* < 0.001).

Having successfully established the epigenetic repression of *RUNX2* and *OSX* in PDL cells, we further examined the role of H3K27me3 histone modification in periodontal ligament mineralization in a rat maxilla model system. PLGA microspheres, in combination with a collagen sponge, were used as delivery media to provide a sustained release of DZNep inhibitor within the periodontal region of first maxillary molars in mice. The morphology of the periodontal structures following 3 weeks of inhibitor treatment was assessed by µCT analysis. Three-dimensional reconstruction revealed a great extent of alveolar bone remodeling at the surgical site in both the control and the DZNep inhibitor-treated rat maxilla. To circumvent any surgery-related artifacts, we conducted our analysis on the distobuccal root of the first molar. The periodontal ligament space was clearly visible and well preserved in both inhibitor-treated and vehicle-treated samples (Figure 5A,B). However, in the case of DZNep-treated samples, the width of the nonmineralized ligament around the root was highly variable, with several regions displaying a considerable decrease in PDL width when compared with control samples (Figure 5B). The PDL width was most affected from the mid-root region running till the root base in inhibitor-treated samples (Figure 5B). The quantification of average PDL width between vehicle and inhibitor-treated samples revealed a statistically significant difference (vehicle treated: 116 µm vs. inhibitor treated: 85 µm; *p* < 0.001) (Figure 5E). Furthermore, the 3D-reconstruction-based computation of total PDL volume around the distobuccal root indicated a ~41% reduction of tissue volume (TV) (*p* < 0.001) in DZNep-treated maxilla compared with controls (Figure 5F).

A histology analysis that was based on trichrome and von Kossa staining further unraveled the differences in cellular architecture and mineral composition between the control and inhibitor-treated samples. While treated control samples displayed a uniform alignment of fibers along the PDL length (Figure 5G), this was lost in the case of DZNep-treated samples, where the fibers were aligned in multiple directions (Figure 5H). Another striking observation was the frequent occurrence of bony outgrowths in the PDL–alveolar bone interface of inhibitor-treated samples (Figure 5H). Von Kossa staining corroborated our findings, with trichrome staining revealing a drastic disruption of the PDL–alveolar bone interface and the presence of mineralized outgrowths within the ligament space of inhibitor-treated samples (Figure 5J) compared with controls (Figure 5I).

## 4. Discussion

In the present study, we used the periodontal region with its extreme contrasts between adjacent mineralized and soft tissues as a model system to assess the effect of the histone methylation-mediated modulation of the chromatin state on periodontal mineral homeostasis. Our ChIP assays revealed unique epigenetic signatures for promoters of key osteogenic transcription factors in periodontal tissues of different mineralization states, associated with differences in osteogenic differentiation levels and mineralized tissue formation. Specifically, *RUNX2* and *OSX* promoters in AB progenitors were preferentially enriched for H3K4me3 histone marks in comparison to PDL progenitors, which demonstrated higher enrichment for H3K27me3 and H3K9me3 marks on both promoters. Gene-expression analysis further corroborated our epigenetic assays, indicating significantly higher *RUNX2* and *OSX* transcript and protein levels in AB progenitors compared with PDL progenitors. Under osteoinductive conditions, alveolar bone cells responded with a linear increase in the H3K4me3 mark on the *RUNX2* promoter, while in periodontal ligament progenitors, the active H3K4me3 mark on the *RUNX2* promoter exhibited a steep increase within the first 7 days and a rapid decline after 14 days. In contrast, 14 days of osteoinductive conditions resulted in a continuous decrease in H3K4me3 and H3K27me3 marks on the *OSX* promoter of both PDL and AB cells. Treatment with histone methylation inhibitors for H3K9me3 and H3K27me3 increased the mineralization response in PDL cells by activating *RUNX2* and *OSX* expression. Furthermore, DZNep-mediated H3K27me3 inhibition led to aberrant mineralization and a decrease in periodontal ligament width. Data obtained in our analysis provide a detailed portrait of the dynamic chromatin state regulating dental progenitor differentiation in that they fine-tune the mineralization control in the periodontal region.

As mentioned in our introduction, mineralized state homeostasis is controlled by two major zinc finger transcription factors, *RUNX2* and *OSX* [38]. In our ChIP studies, alveolar bone cells were characterized by higher levels of active H3K4me3 marks on both *RUNX2* and *OSX* promoters, while PDL progenitors demonstrated higher levels of repressive H3K9me3 and H3K27me3 marks. These data suggest that the reduced level of mineralization in periodontal ligament cells is the result of the key mineralization transcription factor inhibition on a promoter level through changes in histone dynamics. While opposing histone modification marks at the same promoter region might seem perplexing, the presence of functional levels of both active and repressive marks is key to the lineage and differentiation potential of undifferentiated PDL and AB cells. These promoters are therefore poised to be rapidly activated or repressed upon stimulation by environmental cues. In addition, this promoter methylation pattern allows for basal levels of *RUNX2* and *OSX* gene expression, which is higher in the case of lineage-committed AB cells compared with the less-differentiated PDL cells.

Confirming the shutdown of mineralization lineage differentiation mechanisms in periodontal lineages on a transcriptional level, our studies revealed that both *RUNX2* and *OSX* transcripts were reduced in PDL progenitors, while both transcription factors were strongly expressed in alveolar bone cells, resulting in distinct *RUNX2* and *OSX* protein levels in alveolar bone cells and a lack thereof in PDL progenitors. The fidelity of our assay was validated by the detection of similar levels of mineralization matrix gene *IBSP* and the early mineralization marker *ALP* in both PDL progenitors and alveolar bone cells. The preferred expression of *RUNX2* and *OSX* in alveolar bone cells was further confirmed in our immunoreactions, reporting strong *RUNX2* expression in the bone-lining cells of developing alveolar bone and *OSX* expression throughout the periodontal ligament. This finding suggests that *RUNX2* is involved in the spatial confines of alveolar bone shape, while the repression of *OSX* is directly related to the mineralized state of the periodontal ligament. These findings are in congruence with earlier studies [26,39], establishing that periodontal lineage cells as mineralized state–committed neural crest derivatives feature characteristic transcriptional signatures of intermediate precursor populations. Our present study provides further evidence that the gene-expression pattern of committed odontogenic lineages is differentially regulated through histone methylation states on a promoter level, not unlike earlier findings related to dental papilla and dental follicle terminal differentiation [28].

Our mineralization-induction studies provided additional insights into the different modes by which *RUNX2* and *OSX* modulate PDL cell and alveolar bone mineralization. These studies demonstrated that *RUNX2* control is mediated through an increase in H3K4me3 marks, while *OSX* regulation featured a decrease in H3K27me3 repressive marks as the prevailing trend. This finding suggests that the developmental control of periodontal mineralized state inhibition occurs through two somewhat functionally redundant but fundamentally different mechanisms of transcriptional control. Together, they testify to the sophistication and high level of transcriptional fine-tuning of the gene-expression specification in the periodontal region.

In vitro studies using histone methylation inhibitors DZNep (for H3K27me3) and Chaetocin (for H3K9me3) reactivated *RUNX2* and *OSX* expression in periodontal progenitors and increased mineralization indicators, alkaline phosphatase, and Alizarin Red, while the application of DZNep in a rat maxilla model resulted in aberrant mineralization in the periodontal ligament and an encroachment of the mineralized alveolar bone/cementum region onto the nonmineralized periodontal space. Together, these findings provide experimental proof for the epigenetic nature of the periodontal ligament mineralized state inhibition through the repression of *RUNX2* and the repression of *OSX* in periodontal progenitors.

The concept that the nonmineralized state of periodontal tissues is a secondarily evolved state during the course of vertebrate evolution, secondarily to tooth ankylosis as the primary condition, has been documented earlier by our group [14,40,41]. We speculate that this state, gradually acquired over the course of millions of years of vertebrate evolution, has evolved through changes in multiple subsets of gene families and that the control of odontogenic cell fate and differentiation through key transcription factors, *RUNX2* and *OSX*, plays a key role in this process. While *OSX* and *RUNX2* both control cell fate and lineage specification and affect collagen gene expression, *OSX* exerts its regulatory function through the binding to guanine-rich sequences at specific target genes, while *RUNX2* binds to specific DNA sequences at the promoter regions of its target genes [42], ensuring the redundancy of mineralized state control through independent mechanisms. When applying our findings to a macroevolutionary scenario such as the evolution of gomphosis-type attachment, our study revealed that the application of two simple methylation inhibitors in this study partially restored the nonmineralized state of the ligamentous periodontal attachment to its premammalian evolutionary ankylosed state. Our studies further specified that H3K27me3 inhibition alone elevated *ALP* levels in PDL cells in the absence of osteoinductive conditions, suggesting that the changes in the H3K27me3 mark resulted in a major epigenetic switch responsible for *RUNX2* and *OSX* transcriptional repression and, consequentially, mineralization inhibition in these cells. This finding is potentially of evolutionary significance in that *RUNX2* and *OSX* are highly conserved across multiple species [43]. On the basis of the findings of the present study, we propose that repressive histone tail modifications represent a potential epigenetic mechanism responsible for the determination of periodontal ligament nonmineralized state control in nonankylosed tooth-bearing mammals through the repression of master transcription factors *RUNX2* and *OSX*.

## Data Availability

Data are held at the TAMU Center for Craniofacial Research and Diagnosis and are available upon request.

## Supplementary Materials

The following supporting information can be downloaded at https://www.mdpi.com/article/10.3390/genes14010201/s1, Supplemental Table S1: Primer pairs for Real-Time PCR and ChIP analysis.
