# Supplementary material for: Epigenetic Repression of RUNX2 and OSX Promoters Controls the Nonmineralized State of the Periodontal Ligament
Source: Genes (Basel). 2023 Jan 12;14(1):201. doi: 10.3390/genes14010201 (PMC9858805; doi:10.3390/genes14010201)
Supplement: Supplementary file 1 [file genes-14-00201-s001.zip › genes-2109933-SI.pdf]

Supplemental Table S1

RT-qPCR AND CHIP OLIGOS USED IN THE STUDY

OLIGOS FOR *RUNX2* and *OSX* PROMOTER ChIP

RUNX2

ChIP A	Forward	GAATCGCTTCTCGGAGTCAC
	Reverse	TTTCCCACGTTTCTCTCCAC
ChIP B	Forward	GCAGGAACGTGGAGAGAAAC
	Reverse	TCCTTCCTAATTGGGGCTTT
ChIP C	Forward	CCCAAGCTCATCTTGTACTCG
	Reverse	CCTAGAAGGGGCCTGGAA
ChIP D	Forward	GCGTTTGCACTGAGCAATAA
	Reverse	CCTCGAAGCATCAAGGAAGA
ChIP E	Forward	CGCAAACACGTTTTCAAGC
	Reverse	GGAAATCCGCTTGAGGCTAT
ChIP F	Forward	CAGAGTTTGAGGCTGGTCGT
	Reverse	TTTGGAGGCTGGGATTTCTA
ChIP G	Forward	TTTAATTTTTCCCCGTGAGC
	Reverse	AAGTTGCAAAACCCTTGCTG
ChIP H	Forward	ACCCAAACCCTGCAAAATCT
	Reverse	TTCTTGAGCCTCTGTGCTGA

OSX

ChIP A	Forward	TGTGTGTCCGTCTGTGTTCA
	Reverse	GAGAGACTGGAACGCACTGA
ChIP B	Forward	CTCCACCTCAGAACCTTCCA
	Reverse	CAGTCACCCACCTGCCTCTA
ChIP C	Forward	GGCCTAGGTATCACCTCCAA
	Reverse	GAGGTTGGTTCCAGAGCAAG
ChIP D	Forward	TTGGTTACCCAGGGATGGTA
	Reverse	GTGGCGGTAGAAGGTCATTG
ChIP E	Forward	TTTGGGTTTCCCTCCTAACC
	Reverse	TCTCATTGCCATGCATCAAT
ChIP F	Forward	GGGCTTTATTCCCCTGACA
	Reverse	GAGGACTGGGCAGCTAAGTG
ChIP G	Forward	TCTATCAGCCACCTGGTTCC
	Reverse	CAGCAGGTAGGCACCAATTT
ChIP H	Forward	TCCCCATCTTTGTTCCTCTG
	Reverse	GAAGACCAGATGTGTGGCTTTA

OLIGOS FOR REAL TIME PCR ANALYSIS		
BMP2	Forward	TCAAGCCAAACACAAACAGC
	Reverse	AGCCACAATCCAGTCATTCC
RUNX2	Forward	GTGCCTAGGCGCATTTCA
	Reverse	GCTCTTCTTACTGAGAGTGGAAGG
OSX	Forward	TACCCCATCTCCCTTGACTG
	Reverse	GCAACAGGGGATTAACCTGA
IBSP	Forward	AACCTACAACCCCACCACAA
	Reverse	CGTACTCCCCCTCGTATTCA
ALP	Forward	CAACCCTGGGGAGGAGAC
	Reverse	GCATTGGTGTTGTACGTCTTG
GAPDH	Forward	ACAGTCAGCCGCATCTTCTT
	Reverse	ACGACCAAATCCGTTGACTC
